# PET Imaging of Crossed Cerebellar Diaschisis after Long-Term Cerebral Ischemia in Rats

**DOI:** 10.1155/2018/2483078

**Published:** 2018-12-02

**Authors:** Ana Joya, Daniel Padro, Vanessa Gómez-Vallejo, Sandra Plaza-García, Jordi Llop, Abraham Martín

**Affiliations:** ^1^Experimental Molecular Imaging, Molecular Imaging Unit, CIC biomaGUNE, San Sebastian, Spain; ^2^Magnetic Resonance Imaging, Molecular Imaging Unit, CIC biomaGUNE, San Sebastian, Spain; ^3^Radiochemistry and Nuclear Imaging, Molecular Imaging Unit, CIC biomaGUNE, San Sebastian, Spain; ^4^Achucarro Basque Center for Neuroscience, Leioa, Spain; ^5^Ikerbasque Basque Foundation for Science, Bilbao, Spain

## Abstract

Crossed cerebellar diaschisis (CCD) is a decrease of regional blood flow and metabolism in the cerebellar hemisphere contralateral to the injured brain hemisphere as a common consequence of stroke. Despite CCD has been detected in patients with stroke using neuroimaging modalities, the evaluation of this phenomenon in rodent models of cerebral ischemia has been scarcely evaluated so far. Here, we report the *in vivo* evaluation of CCD after long-term cerebral ischemia in rats using positron emission tomography (PET) imaging with 2-deoxy-2-[^18^F]fluoro-D-glucose ([^18^F]FDG). Imaging studies were combined with neurological evaluation to assess functional recovery. In the ischemic territory, imaging studies showed a significant decrease in glucose metabolism followed by a progressive recovery later on. Conversely, the cerebellum showed a contralateral hypometabolism from days 7 to 14 after reperfusion. Neurological behavior showed major impaired outcome at day 1 after ischemia followed by a significant recovery of the sensorimotor function from days 7 to 28 after experimental stroke. Taken together, these results suggest that the degree of CCD after cerebral ischemia might be predictive of neurological recovery.

## 1. Introduction

Crossed cerebellar diaschisis (CCD) is a decrease of regional blood flow and glucose metabolism in the cerebellar hemisphere contralateral to the affected brain hemisphere as a common consequence of a supratentorial cerebral malfunction [1–3]. Previous studies have suggested that the remote deactivation of cerebellar neurons can be promoted as a result of the interruption of excitatory impulses by the corticopontocerebellar tract [4]. CCD has been reported in several diseases affecting the brain such as cerebral gliomas [5], epilepsy [6], intracerebral hemorrhage [7, 8], and stroke [3, 9–11], among others. In the latter, CCD has been detected at the acute, subacute, and chronic phases. As a result, CCD has been postulated as a prognostic indicator of neurological outcome after cerebrovascular diseases [12, 13]. Nevertheless, other studies have evidenced controversies regarding the correlation of CCD to the location and size of the brain injury and to the clinical severity [14, 15]. In view of these controversial results, the use of *in vivo* imaging techniques acquires a relevant role in the investigation of the precise role of CCD on stroke pathophysiology. CCD in human supratentorial brain infarction has been detected with positron emission tomography (PET) and single photon emission computed tomography (SPECT) [13, 14, 16–19]. Alternatively to nuclear imaging techniques, arterial spin-labeling (ASL) has also proven efficient for the evaluation of CCD after hyperacute and acute ischemic stroke [9, 20]. Unexpectedly, although CCD has been detected in patients with stroke, the *in vivo* imaging evaluation of this phenomenon in rodent models of cerebral ischemia has been never evaluated so far. Here, we report the unprecedented investigation of CCD after long-term cerebral ischemia in a rat model of experimental stroke using PET with 2-deoxy-2-[^18^F]fluoro-D-glucose ([^18^F]FDG). Glucose metabolic changes in the ischemic brain (cerebrum, cortex, and striatum) were also investigated, and the results were correlated to the neurological outcome evolution over time. Our studies show contralateral cerebellar hypometabolism at the acute and subacute phases of ischemia and a correlation between the presence of CCD and the improvement of neurological outcome. Hence, these results reported here will provide novel information on the detection of the CCD in experimental stroke that might ultimately contribute to a better comprehension of the role of this phenomenon during stroke evolution.

## 2. Materials and Methods

### 2.1. Cerebral Ischemia

9-week-old male Sprague-Dawley rats (*n*=6; 295 ± 6.2 g body weight; Janvier, France) were used for imaging studies. Rats were anaesthetized with 2.5% isoflurane in 100% O_2_ and transient focal ischemia was produced by a 2-hour intraluminal occlusion of the middle cerebral artery (MCAO) followed by reperfusion as described previously [21]. Six rats were repeatedly scanned before reperfusion (day 0) and at 1, 3, 7, 14, 21, and 28 days after ischemic onset to evaluate glucose metabolism by PET.

### 2.2. Magnetic Resonance Imaging

T2-weighted (T_2_W) MRI scans were performed at days 1 (to measure the size of the infarction; *n*=6), 3, 7, 14, 21, and 28 after MCAO (to coregister PET signal data; *n*=1 per time point). Before the scans, anesthesia was induced with 4% isoflurane and maintained by 2–2.5% of isoflurane in 30% O_2_/70% N_2_ during the scan. Animals were placed into a rat holder compatible with the MRI acquisition systems and maintained normothermia using a water-based heating blanket at 37°C. MRI experiments were performed on a 7 Tesla Bruker Biospec 70/30 MRI system (Bruker Biospin GmbH, Ettlingen, Germany), interfaced to an AVANCE III console. The BGA12-S imaging gradient (maximum gradient strength 400 mT/m switchable within 80 *µ*s), an 82 mm inner diameter quadrature volume resonator for transmission, and a surface rat brain coil for reception were used. T_2_W images were acquired with a RARE sequence with the following parameters: RARE factor 2, TR/TE = 4400/40 ms, FOV = 25 mm × 25 mm, ACQ Matrix = 256 × 256, Slice Thickness = 1 mm, 2 averages, and 24 contiguous slices.

### 2.3. Magnetic Resonance Imaging Image Analysis

MRI (T_2_W) images at 1 day after ischemia were used to calculate the lesion volume. Regions of interest (ROIs) were manually defined using the open source software 3D Slicer image analysis software (Version 4.8 http://www.slicer.org) for each rat on the region of increased signal in the ipsilateral hemisphere. The total lesion volume was calculated by summing the area of the infarcted regions of all slices affected by the lesion.

### 2.4. Radiochemistry, Positron Emission Tomography Scans, and Data Acquisition

[^18^F]-2-fluoro-2-deoxy-D-glucose ([^18^F]FDG) was produced as described earlier [22] and provided by IBA Molecular Spain (San Sebastian, Spain). PET scans were performed using a General Electric eXplore Vista CT camera (GE Healthcare). Scans were performed in rats anaesthetized with 4% isoflurane and maintained by 2–2.5% of isoflurane in 100% O_2_. The tail vein was catheterized with a 24-gauge catheter for intravenous administration of the radiotracer. For longitudinal assessment of glucose metabolism with [^18^F]FDG, animals were scanned before and during the following month after ischemia. The radioactivity (∼10 MBq) was injected and after an uptake period of 30 minutes, the animals were reanaesthetized and placed on the PET for a brain static acquisition in the 400–700 keV energetic window, with a total acquisition time of 30 minutes as described elsewhere [23]. After each PET scan, CT acquisitions were also performed (140 *µ*A intensity, 40 kV voltage), to provide anatomical information of each animal as well as the attenuation map for the later PET image reconstruction. Static acquisitions were reconstructed (decay and CT-based attenuation corrected) with filtered back projection (FBP) using a ramp filter with a cut-off frequency of 0.5 mm^−1^.

### 2.5. Positron Emission Tomography Image Analysis

PET imaging analysis was performed according to our previously published procedure [24]. PET images were analyzed using PMOD image analysis software (PMOD Technologies Ltd, Zürich, Switzerland). To verify the anatomical location of the signal, PET images were coregistered to the anatomical data of a MRI rat brain template. Two type of volumes of interest (VOIs) were established as follows: (i) A first set of VOIs was defined to study the whole brain and cerebellum [^18^F]FDG PET signal. Whole brain and cerebellum VOIs were manually drawn in both the entire ipsilateral and contralateral hemispheres on slices of a MRI (T_2_W) rat brain template from the PMOD software. (ii) A second set of VOIs was automatically generated in the cortex and the striatum by using the regions proposed by the PMOD rat brain template, to study the evolution of [^18^F]FDG PET signal in these specific regions in both the ipsilateral and contralateral cerebral hemispheres. PET signal uptake was averaged in each ROI and expressed as percentage of injected dose per cubic centimetre (%ID/cc), and the hemispheres ratios were considered.

### 2.6. Neurological Assessment

The assessment of neurological outcome induced by cerebral ischemia was based on a previously reported 9-neuroscore test [25]. Before imaging evaluations, four consecutive tests were performed at days 1 and 7 after ischemia in treated and control rats as follows: (a) spontaneous activity (moving and exploring = 0, moving without exploring = 1, no moving = 2); (b) left drifting during displacement (none = 0, drifting only when elevated by the tail and pushed or pulled = 1, spontaneous drifting = 2, circling without displacement or spinning = 3), (c) parachute reflex (symmetrical = 0, asymmetrical = 1, contralateral forelimb retracted = 2), and (d) resistance to left forepaw stretching (stretching not allowed = 0, stretching allowed after some attempts = 1, no resistance = 2). Total score could range from 0 (normal) to a 9 (highest handicap) point-scale.

### 2.7. Statistical Analyses

PET imaging comparisons within ischemic group were made with one-way ANOVA followed by Tukey's multiple-comparison tests for post hoc analysis. Behavioral data have been compared with Mann–Whitney *U* tests. The level of significance was regularly set at *P* < 0.05. Statistical analyses were performed with GraphPad Prism version 6 software.

## 3. Results

**Figure 1:**
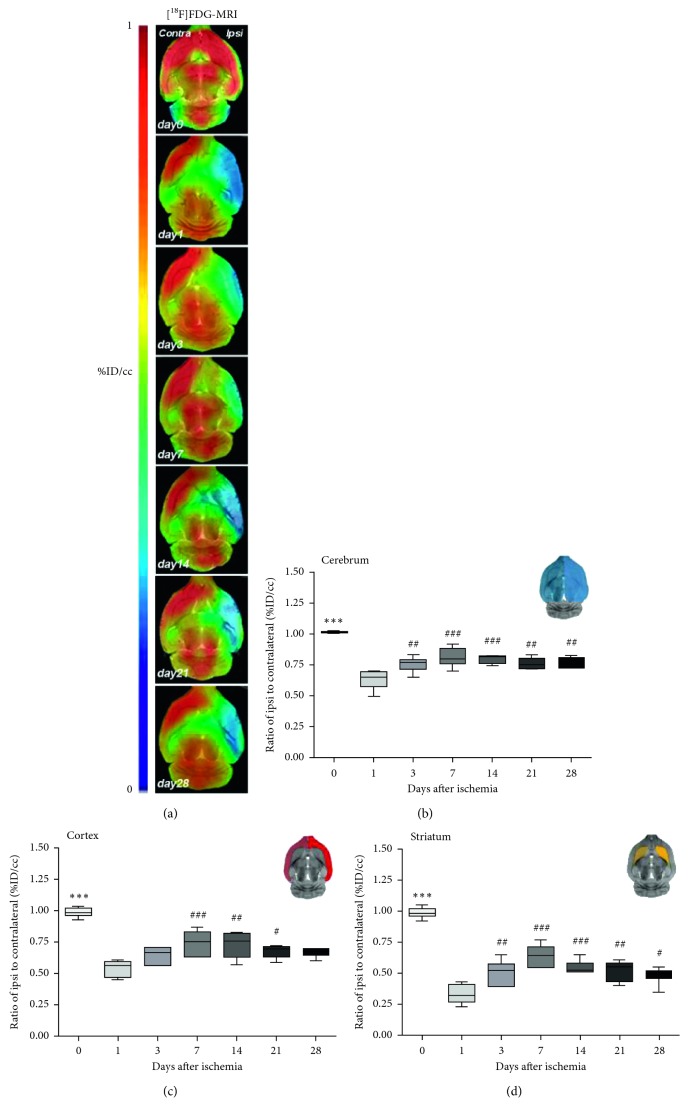
Serial images of [18F]FDG PET following cerebral ischemia. Normalized coronal PET images of [^18^F]FDG at day 0 (control), day 1, day 3, day 7, day 14, day 21 and day 28 after MCAO are coregistered with the MRI (T2W) to localize anatomically the PET signal (a). Images correspond to the lesion evolution of the same animal over time. Time course of the progression of the [^18^F]FDG PET signal (*n*=6) was shown as the ratio of the entire ipsilateral to contralateral cerebral hemisphere (cerebrum) (b), cortex (c) and striatum (d). The upper right panels of each figure show the selected brain ROIs for the quantification defined on a slice of a MRI (T_2_W) template. ^*∗∗∗*^*p* < 0.001 compared to control, ^#^*p* < 0.05, ^##^*p* < 0.01 and ^###^*p* < 0.01 compared to day 1.

The cerebral glucose metabolism was explored by PET imaging during the first month after transient focal ischemia in rats. All the images were quantified in standard units, i.e., %ID/cc of [^18^F]FDG. The images with normalized color scale illustrate the evolution of the PET signals at control (day 0) and at 1, 3, 7, 14, 21, and 28 days after ischemia onset (Figure 1). The extent of brain damage after cerebral ischemia was assessed using T_2_W MRI at 1 day after reperfusion. Hyperintensities of T_2_W images showed similar infarct extents as well as locations affected. All ischemic rats subjected to nuclear studies showed cortical and striatal MRI alterations (mean ± s.d.: 296.24 ± 48.3 mm^3^, *n*=6).

### 3.1. [^18^F]FDG after Cerebral Ischemia

The time course of the cerebral glucose metabolism was evaluated with [^18^F]FDG in both the ipsilateral and contralateral cortexes, striatum, and whole brain at control and 1, 3, 7, 14, 21, and 28 days after MCAO (Figure 1, *n*=6). The different regions evaluated showed similar metabolic evolution after long-term focal cerebral ischemia. In the whole brain (cerebrum), the ratio of the [^18^F]FDG signal uptake in the ipsilateral to contralateral hemispheres showed a general metabolic decrease in the ipsilateral hemisphere after cerebral ischemia in relation to control (day 0) values (*p* < 0.001, Figure 1(b)). At day 1, the [^18^F]FDG signal ratio showed the lowest values in comparison to day 0 (control) followed by a progressive increase from day 3 to days 7–14 after ischemia in comparison to day 1 (*p* < 0.01; *p* < 0.001, Figure 1(b)). Subsequently, the PET signal ratio displayed a slight decrease from days 21 to 28 after ischemia onset (*p* < 0.01, Figure 1(b)). The cerebral cortex showed a significant reduction of the [^18^F]FDG-PET ratios from 1 at day 0 to 0.5 at day 1 after reperfusion followed by a recovery to circa 0.7 at days 7 and 14 and a subsequent decrease later on (days 21–28) (*p* < 0.05; *p* < 0.01; *p* < 0.001, with respect to control and day 1, Figure 1(c)). In addition, the striatum showed similar [^18^F]FDG-PET signal values over the first moth after cerebral ischemia than that observed in the whole brain and cerebral cortex (*p* < 0.05; *p* < 0.01; *p* < 0.001, with respect to control and day 1, Figure 1(d)).

### 3.2. Crossed Cerebellar Diaschisis after Ischemia

**Figure 2:**
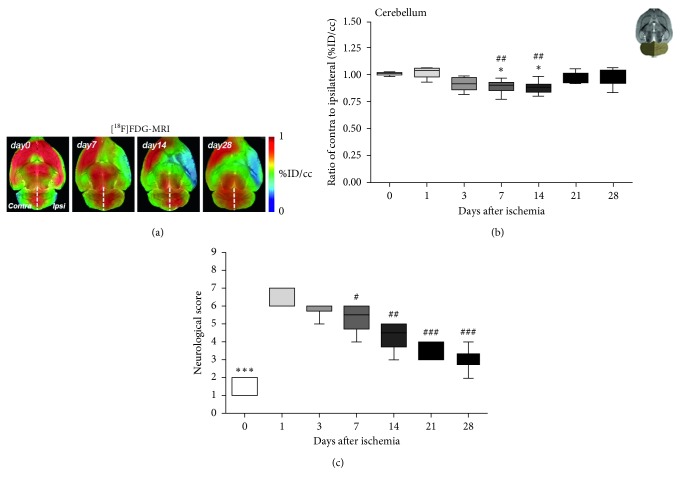
PET images of [^18^F]FDG at day 0 (control), day 7, day 14 and day 28 after MCAO (a). Time course of the progression of the [^18^F]FDG PET signal (*n*=6) was shown as the ratio of the entire contralateral to ipsilateral cerebellar hemisphere. The upper right panel of the figure shows the selected brain ROIs for the quantification defined on a slice of a MRI (T2W) template (b). Neurologic outcomes before (day 0) and at 1, 3, 7, 14, 21 and 28 days after cerebral ischemia (c). ^*∗∗∗*^*p* < 0.001 compared to control, ^#^*p* < 0.05, ^##^*p* < 0.01 and ^###^*p* < 0.01 compared to day 1.

The time course of the glucose metabolism in the cerebellum was evaluated with [^18^F]FDG at the acute, subacute, and chronic stages after MCAO (Figure 2, *n*=6). In the cerebellum, the ratio of the contralateral to ipsilateral hemispheres showed a progressive glucose metabolism decrease from control (day 0) to day 3 followed by a significant decrease at days 7 and 14 in comparison to day 0 and day 1 after cerebral ischemia. Therefore, these results evidence the existence of CCD after cerebral ischemia in rats (*p* < 0.05; *p* < 0.01, Figure 2(b)). Subsequently, the [^18^F]FDG signal ratios displayed a recovery to control values at days 21 and 28 after MCAO. The time course of the neurologic score after cerebral ischemia showed the major neurologic impairment at day 1 after MCAO in relation to control rats followed by a progressive significant improvement from days 7 to 28 after ischemia (*p* < 0.05; *p* < 0.01; *p* < 0.001, with respect to control and day 1, Figure 2(c)).

## 4. Discussion

Since Baron and collaborators coined the term CCD in the 1980s to describe the disturbance of the cerebellum distant but linked to an ischemic region of the brain, the pathophysiology underlying this phenomenon has been still plenty of controversy [3, 10]. Some clinical studies in stroke have defined diaschisis as a transient and reversible event attributed to a functional neuronal deafferentation. However, others have described CCD as a persistent process that leads to neurodegeneration [18, 26–28]. Likewise, CCD has been extensively diagnosed clinically with neuroimaging modalities in patients at different stages after stroke [9, 11, 13, 29–31]. Despite this, CCD has remained poorly characterized in animal models of stroke. Because of this, we have assessed for the first time the *in vivo* imaging of CCD using PET with [^18^F]FDG imaging in combination to neurofunctional evaluation after cerebral ischemia in rats.

### 4.1. [^18^F]FDG-PET Imaging after Cerebral Ischemia

Contralateral cerebellar hypometabolism (CCH) is a well-established remote functional effect related to CCD that might be promoted by the uncoupling of the oxygen consumption and glucose utilization caused by neuronal deafferentation [32]. Moreover, it is known that astrocytes respond to neuronal deafferentation and a recent report indicates that [^18^F]FDG signal is sensitive to astrocyte metabolism suggesting the potential role of astrocytes on CCH [33, 34]. Contralateral cerebellar hypometabolism has been observed clinically with [^18^F]FDG-PET imaging after stroke; however, the correlation to the clinical significance is still under debate [12]. Therefore, the preclinical characterization of the CCH in animal models of cerebral ischemia with [^18^F]FDG might provide novel perspectives to the understanding of CCD after stroke. In the present study, we have assessed the characterization of the CCH during the first month following cerebral ischemia in rats by using [^18^F]FDG. After experimental stroke, rats showed a decreased [^18^F]FDG uptake in the region of the infarction followed by a progressive recovery during the first week after ischemia onset (Figure 1). In fact, the increase of glucose metabolism at day 7 stands in agreement with the activation of microglial cells and infiltrated leukocytes after cerebral ischemia [24, 35]. Subsequently, [^18^F]FDG PET showed a progressive decrease of the glucose metabolism from day 14 to 28 due to a (i) reduction of the inflammatory response and (ii) the reabsorption of the necrotic cerebral tissue [36, 37]. In addition, as a result of the supratentorial ischemic lesion, PET imaging with [^18^F]FDG displayed a contralateral cerebellar hypometabolism from days 7 to 14 after MCAO that was followed by a recovery to control values during the second and the third week later on (Figure 2). In addition, we have previously demonstrated that [^18^F]FDG signal did not show any change over one month in both control and SHAM 9-week-old male Sprague-Dawley rats [23]. Therefore, these results evidence the existence of CCD after acute and subacute stages of cerebral ischemia in rats that are reverted at the chronic phase and are in agreement with the reversibility of the CCH in some patients with stroke [12]. Nevertheless, despite only one-third of the patients in the acute phase showed CCD after large anterior circulation vessel occlusion [31, 38], all rats evaluated in this study presented cerebellar diaschisis. Likewise, the ischemic rats included in this study showed a very similar location and size of lesion (296.24 ± 48.3 mm^3^). However, the extension of the lesion did not show a significant correlation to the CCH observed at days 7 and 14 after MCAO (data not shown). Therefore, these results are in disagreement with those described by Infield and colleagues who defined CCD as the functional phenomenon that correlates with the severity of the ischemic lesion [15]. In the present study, the neurological score showed that the animals presented the worst outcome at the day 1 after ischemia followed by a progressive significant functional improvement from day 7 to onwards. Therefore, the neurological recovery experienced by the ischemic rats run in parallel with the presence of the CCD at the second and third week after cerebral ischemia. Likewise, these findings stand in contrast with the description of cerebellar diaschisis as the phenomenon that can persist despite the recovery of neuronal functionality [15].

In summary, PET imaging with [^18^F]FDG was carried out to evaluate the CCD after cerebral ischemia in rats. Our studies showed for the first time the contralateral cerebellar hypometabolism at the acute and subacute phases of cerebral ischemia in rats. Besides, the presence of CCD stands in agreement with the improvement of neurological outcome. Therefore, these results provide valuable knowledge regarding the role of CCD after experimental stroke and suggest that the degree of cerebellar diaschisis might be predictive of neurological recovery in rats.

## Data Availability

All data supporting the results can be found at CICbiomaGUNE, San Sebastian, Spain, or from the corresponding author upon request.
